# Physical fitness in preschool children in relation to later body composition at first grade in school

**DOI:** 10.1371/journal.pone.0244603

**Published:** 2021-01-13

**Authors:** Kirkke Reisberg, Eva-Maria Riso, Jaak Jürimäe

**Affiliations:** 1 Institute of Sports Sciences and Physiotherapy, Tartu University, Tartu, Estonia; 2 Tartu Healthcare College, Tartu, Estonia; The Education University of Hong Kong, HONG KONG

## Abstract

**Background:**

This study aimed to investigate whether better physical fitness in kindergarten predicts later healthier body composition in first grade at school.

**Methods:**

Body composition was assessed by skinfold thickness measurements. Physical fitness tests included 20 m shuttle run test, handgrip strength test, standing long jump test, 4x10 m shuttle run test as part of PREFIT fitness test battery, and one-leg stance test from EUROFIT test battery. The participants of this study were 147 Estonian children (51% boys) aged 6–8 years, who were measured in the transition from kindergarten to school.

**Results:**

After adjusting for maternal body mass index, educational attainment, child’s sex, age at the measurements, greater cardiovascular and motor fitness, relative lower body strength, static balance at 6.6 yr were associated with lower fat mass index, fat mass percentage at 12-month follow-up. The relative lower body strength above the median at 6.6 yr were related to lower fat mass index and fat mass percentage at 12-month follow-up, while the static balance test results demonstrated the opposite associations. Improvements in the 4x10 m shuttle run test results during the 12-month follow-up period were associated with the most beneficial changes in body composition status, such as increases in fat-free mass index and decreases in fat mass index, fat mass percentage, waist-to-height ratio after adjusting for maternal body mass index, educational attainment, child’s sex, age, at the measurements and baseline values of exposures.

**Conclusion:**

Better physical fitness tests results at 6.6 yr in kindergarten generally predicted lower body fat parameters in children at 7.6 yr in first grade at school.

## Introduction

Physical fitness (PF) [1] and body composition [2] both can be regarded as indicators of health and well-being already at young age. Increasing rate of overweight and obesity in peadiatric population is alarming health concern with serious psychosocial and economic consequences [3, 4]. Excess adiposity is the main risk factor for metabolic syndrome (including abdominal obesity, insulin resistance, dyslipidemia, hypertension) [5] and overweight children are more prone to prematurely developing type 2 diabetes [6]. Both obesity and hypertension are linked to increased cardiovascular disease risk tracking into adulthood [7].

Furthermore, there is some evidence that obesity might have an impact on academic achievement in children mediated by others attitude and diminished executive cognitive functions [8], and obese adolescent girls have further lower educational attainment and income [9]. Obesity is associated with greater prevalence of depression among girls, with preserved risk in adulthood [10].

Taking into consideration that even short periods of overweight/obesity during childhood increase the risk of earlier mortality in adulthood [11] and that childhood obesity has strong tendency for long-term persistence [12], early detection of appropriate public lifestyle interventions is of major importance. We are aware that kids who are physically fit have better physical and mental status [1], but less is known whether body composition at school could be possibly mediated by PF level at kindergarten. Adding this information could further augment our understanding about the longer-term effects of PF on health indicators in childhood. Accordingly, the primary objective of this longitudinal study was to determine whether greater PF in kindergarten predicts healthier body composition during the transition to school.

## Materials and methods

### Data collection and participants

A two-phased longitudinal study was conducted, assessing the associations between PF level at kindergarten with the body composition one year later after at first grade in school. During the first period of study, children who were in their final year of kindergarten, were recruited from 13 randomly chosen kindergartens in the city of Tartu, and nearby regions, in Estonia. The parents/caregivers of children from 400 families were invited to take part in the study and were provided with written information about the study. Written consents and assents to participate in the study were given by 284 families. Measurements were made from March to May 2016.

The second study period was conducted one year later, from March to September 2017. The same parents and children who had participated in the first period of study and had now entered at the first grade in school were contacted and asked to take part of the study again. In total, 200 families agreed to participate in the second measurement time. We lost children in second study period due to their relocation to another city or country, and in other cases we can not tell the reasons for refusal to participate, as according to the regulations of Medical Ethics Committee of the University of Tartu participants do not have to state reasons for non-participation. All procedures related to delivering information, obtaining consents, assents, and assessments of children were performed similar to the first period.

Altogether, data from 147 children (51% boys) aged 6.6 and 7.6 yr were analysed after excluding those with missing data. The measurements were performed either in the kindergarten (first period) or school settings (second period). The study was approved by the Medical Ethics Committee of the University of Tartu, Tartu, Estonia (reference 254/T-16).

### Variables

Body height and weight were measured using calibrated medical digital scales (A&D Instruments, Abington, UK) and portable stadiometer (Seca 213, Hamburg, Germany) to the closest 0.05 kg and 0.1 cm, respectively, with the participant wearing light clothing without shoes [13, 14]. Waist circumference was measured twice using a metal tape from the Centurion kit (Rosscraft, Canada), at the level of the narrowest point between the lower costal (10th rib) border and the iliac crest, and if there was no obvious narrowing the measurement was taken at the mid-point between the lower costal (10th rib) border and the iliac crest. Height and waist measurements were performed in triplicate and duplicate, respectively, and in rotational order, the mean of both measurements was used in our analyses [13–17]. Waist-to-height ratio (WHtR) was derived from waist (cm)/height (cm) [14, 16–18]. Body mass index (BMI) was calculated as body mass (kg) divided by body height squared (m^2^). Overweight and obese subjects were defined by age-specific BMI cutoff points [19]. Skinfold thicknesses at the *triceps* and *subscapular* site of the body were measured in triplicate, in rotational order on the right side of the body with a Holtain caliper (Crymmych, UK) to the nearest 0.2 mm based on standardized procedures [15]. In kindergartens, the same trained investigator made all skinfold thickness measurements. Before the measurements in school settings, the members of data collecting team trained the measurements procedures as shown in ISAK protocol [15] until for all the skinfold thickness measurements, intra-observer technical errors were smaller than 1 mm and reliability greater than 95%. Interobserver reliability for skinfolds was higher than 90% [14]. In every school, the same trained investigators made the skinfold thickness measurements. The percentage of body fat (FM%) and fat mass (FM) were calculated from *triceps* and *subscapular* skinfold thicknesses using the equations by Slaughter et al. [20] for 6–17 yr old children and youth. Fat-free mass (FFM) (kg) was derived by subtracting FM from total body mass [21]. Fat mass index (FMI) (kg/m) was calculated as FM (kg) x height (m)^-2^, and fat-free mass index (FFMI) as FFM (kg) x height (m)^-2^ [14, 16, 17, 22, 23].

Standardized PREFIT fitness test battery was used to assess the participants’ PF components, such as cardiorespiratory fitness, upper and lower body muscular strength and motor fitness [13, 24]. Cardiorespiratory fitness was assessed by 20 m shuttle run test, where the participant was required to run between two lines 20 m apart and the running pace was set by the audio signals. The initial speed was 8.5 per hour and increased by 0.5 km per hour every minute. The test was finished when the child could not reach the end lines concurrent with the audio signal on 2 consecutive occasions or when the child stopped because of exhaustion and the number of laps was recorded when scoring the test [13, 17, 23, 25]. Upper body muscular strength was assessed with the handgrip strength test [13, 17, 23], using digital dynamometer (Digital TKK 5401, Grip D, Takei, Tokyo) with adjustable handle, in order to fit for individual use, and the following formula was used to find optimal handle position: y = x/5 + 1.5, where x = hand span measured from the tip of the thumb to the tip of the small finger with the hand opened as wide as possible (cm), and y = the grip span settled for dynamometer. Children squeezed dynamometer gradually as hard as they could for at least 2 or 3 s, and test was performed twice, alternately with both hands. The elbow was held in extended position, avoiding the contact of dynamometer with other parts of the body, except the hand being measured. The best value of the two trials for each hand was taken into consideration, and the mean of both hands was calculated (kg). Lower body muscular strength was assessed by the standing long jump test (SLJ) (cm) [13, 17, 23], in which the child stood behind the line legs opened shoulder width, and had to jump as far as possible with feet together and remaining upright when landing, arm swing was allowed in take-off. Motor fitness was assessed by the 4x10 m shuttle run test (s) [13, 17, 23] where the child had to run and turn as fast as possible between two parallel lines drawn on the floor 10 m apart, covering thus a distance of 40 m. Static balance was assessed by the EUROFIT modified Flamingo balance test (s) [26], child standing on preferred leg on a wooden beam (50x4x3 m), the free leg flexed at the knee avoiding the contact with floor and supporting leg, with the goal to maintain the balanced position as long as possible. The chronometer was activated when the free leg left the floor and stopped when required position could not be maintained. All PF tests were conducted twice, and the best value of two attempts was included into analysis. The only exception was 20 m shuttle run test, that was performed once [13].

Upper and lower body muscular strength were expressed as relative measures, normalizing by BMI [27, 28] and FFM [29, 30]. To calculate the grip-to-BMI ratio and the grip-to FFM ratio, handgrip strength value was divided by BMI and FFM, respectively. In similar way, the SLJ-to-BMI ratio and the SLJ-to-FFM ratio were calculated, by dividing SLJ test result by BMI and FFM, respectively.

### Statistical analysis

The SPSS software (version 20.0; SPSS, Inc., Chicago, IL, USA) was used for the data analysis. Descriptive statistics is given as means, standard deviations (SD) or frequencies (percentages). All variables were checked for normality before the analysis using the Kolmogorov–Smirnoff test. Group differences between the means were explored with the paired t-test, and the chi-square test was used to analyse group differences with categorical values. The variance inflation factors between variables were less than five, denoting that collinearity was not a concern. Linear regression analysis was conducted to investigate the unadjusted and adjusted associations between PF parameters at 6.6 yr with body composition measurements at 1-yr follow-up. Adjustments for potential confounders such as maternal BMI, maternal educational attainment, child’s sex and age, at the measurements, and additionally baseline values of exposures and outcomes in the analysis of change in PF and body composition between the age period of 6.6 to 7.6 yr was applied in adjusted model [31]. The results of the regression models are reported as standardized regression coefficient (β). Analysis of covariance was used in examining whether PF levels above and below the median at 6.6 yr associated with body composition at 1-yr follow-up. In all tests p<0.05 was considered statistically significant.

## Results

### Participants’ characteristics

The characteristics of the 147 children at baseline and at 1-yr follow-up are shown in Table 1. Body composition parameters were greater at 7.6 yr compared to 6.6 yr (all p≤0.049), except for FMI and FM%, where older children had lower values (both p<0.001). The results of all PF tests were better at 7.6 yr compared to 6.6 yr (p<0.001), except for one-leg stance test, where younger age group demonstrated better balance (p<0.001). More than half of children both at 6.6 yr and at 1-yr follow-up (62 and 69.7%, respectively) obtained the recommended 60 min or more moderate-to-vigorous physical activity daily [32].

**Table 1:** Description of sample data at 6.6 yr (kindergarten) and at 7.6 yr (school).

	Baseline	Follow-up	*P* difference
Age (yr)	6.6 ± 0.51	7.63 ± 0.49	**<0.001**
Height (cm)	125 ± 5.77	132 ± 6.33	**<0.001**
Weight (kg)	25.4 ± 4.23	28.7 ± 5.44	**<0.001**
BMI (kg/m^2^)	16 ± 1.68	16.3 ± 2.06	**0.001**
Overweight (%)	13	13	1.000
FFMI (kg/m^2^)	12.6 ± 0.99	13.3 ± 1.07	**<0.001**
FMI (kg/m^2^)	3.4 ± 1.03	3 ± 1.28	**<0.001**
FM (%)	20.9 ± 4.27	17.7 ± 5.08	**<0.001**
WHtR	0.43 ± 0.03	0.44 ± 0.03	**0.049**
Physical fitness tests			
20 m shuttle run (laps)	19.8 ± 9.57	23.7 ± 13.53	**<0.001**
Handgrip strength (kg)	11 ± 2.23	13.6 ± 2.86	**<0.001**
Standing long jump (cm)	122 ± 18.3	134 ± 21.7	**<0.001**
4x10 m shuttle run (s)^*a*^	15.4 ± 2.58	14.7 ± 1.44	**<0.001**
One-leg stance (balance) (s)	21.9 ± 10.4	12.1 ± 8.3	**<0.001**

Data are given as mean ± SD.

^*a*^The lower the score (in seconds), the higher the performance.

Group comparisons were made by using paired t-test, except chi-square test for categorized variables (overweight).

P < 0.05 was considered statistically significant.

BMI—body mass index; FFMI—fat-free mass index; FM—fat mass; FMI—fat mass index; WHtR–waist-to-height ratio.

### Associations of physical fitness at 6.6 yr (kindergarten) with body composition at 7.6 yr (school)

Table 2 demonstrates the associations between PF at the age of 6.6 yr with body composition at 1-yr follow-up. It was found out that better cardiorespiratory fitness at 6.6 yr was associated with lower FMI and FM% at 7.6 yr both in unadjusted models (p = 0.029, p = 0.020, respectively) and also after adjusting for maternal body mass index, maternal educational attainment, child’s sex and age at the measurements (p = 0.002, p<0.001, respectively). We did not identify any associations between cardiorespiratory fitness at 6.6 yr and the rest of body composition parameters at 7.6 yr (p>0.05).

**Table 2:** Associations of physical fitness^*a*^ at 6.6 yr (kindergarten) with body composition at 7.6 yr (school).

	Physical fitness^a^ at 6.6 yr													
Cardiorespiratory fitness			Grip-to-BMI ratio		Grip-to-FFM ratio		SLJ-to-BMI ratio		SLJ-to-FFM ratio		Motor fitness		Balance	
Body composition at 7.6 yr	β	*P*	β	*P*	β	*P*	β	*P*	β	*P*	β	*P*	β	*P*
BMI (kg/m^2^)														
Unadjusted	-0.143	0.121	-0.091	0.320	-0.121	0.200	-0.515	**<0.001**	-0.442	**<0.001**	0.067	0.464	-0.282	**0.002**
Adjusted^b^	0.059	0.103	0.142	0.063	0.076	0.080	-0.332	**0.005**	-0.342	**<0.001**	0.032	0.098	-0.366	**0.003**
FMI (kg/m^2^)														
Unadjusted	-0.200	**0.029**	-0.188	**0.038**	-0.162	0.085	.0.581	**<0.001**	-0.138	0.160	0.113	0.215	-0.251	**0.005**
Adjusted^b^	-0.018	**0.002**	0.080	**0.001**	0.028	**0.003**	-0.373	**<0.001**	-0.192	**<0.001**	0.057	**0.001**	-0.360	**<0.001**
FM (%)														
Unadjusted	-0.213	**0.020**	-0.212	**0.020**	-0.161	0.087	-0.582	**<0.001**	0.006	0.952	0.120	0.188	-0.229	**0.011**
Adjusted^b^	-0.047	**<0.001**	0.045	**<0.001**	-0.009	**<0.001**	-0.373	**<0.001**	-0.166	**<0.001**	0.072	**<0.001**	-0.314	**<0.001**
FFMI (kg/m^2^)														
Unadjusted	-0.036	0.695	0.050	0.586	-0.041	0.668	-0.302	**<0.001**	-0.498	**<0.001**	-0.005	0.956	-0.246	**0.006**
Adjusted^b^	0.132	0.438	0.166	0.364	0.108	0.457	-0.168	0.353	-0.399	**<0.001**	-0.009	0.542	-0.360	**<0.001**
WHtR														
Unadjusted	-0.152	0.100	-0.305	**0.001**	-0.256	**0.004**	-0.508	**<0.001**	-0.381	**<0.001**	0.160	0.076	-0.247	**0.006**
Adjusted^b^	0.035	0.197	-0.257	**0.045**	-0.250	**0.044**	-0.391	**0.003**	-0.209	**<0.001**	0.185	0.091	-0.320	**0.019**

The results are presented as standardized regression coefficient (β), and *P* value are given for each association. *P* < 0.05 was considered statistically significant.

^*a*^Physical fitness was measured using 20 m shuttle run test (cardiorespiratory fitness), handgrip strength test (upper body muscular strength normalized to BMI and FFM: grip-to-BMI ratio, grip-to-FFM ratio, respectively), standing long jump test (lower body muscular strength normalized to BMI and FFM: SLJ-to-BMI ratio, SLJ-to-FFM ratio, respectively), 4×10 m shuttle run test (motor fitness) from the PREFIT battery, and one-leg stance test (balance) from EUROFIT battery.

^*b*^Adjusted for maternal BMI, maternal educational attainment, child’s sex and age, at the measurements.

BMI—body mass index; FFM–fat-free mass; FFMI—fat-free mass index; FM—fat mass; FMI—fat mass index; WHtR–waist-to-height ratio.

Greater grip-to-BMI ratio at 6.6 yr was associated with lower FMI and FM% at 7.6 yr in unadjusted models (p = 0.038, p = 0.020, respectively), but these associations were reversed after adjusting for potential confounding variables (p = 0.001, p<0.001, respectively). In unadjusted (p = 0.001) and adjusted models (p = 0.045), greater grip-to-BMI ratio at 6.6 yr was associated with lower WHtR at 7.6 yr. Grip-to-BMI ratio at 6.6 yr was not associated with BMI or FFMI at 1-yr follow-up. Greater grip-to-FFM ratio at 6.6 yr was associated with greater FMI (p = 0.003), but lower FM% (p<0.001) at 7.6 yr after adjusting for confounders. Greater grip-to-FFM ratio at 6.6 yr was associated with lower WHtR at 7.6 yr in unadjusted (p = 0.004) and adjusted models (p = 0.044). No significant associations were found between grip-to-BMI ratio or grip-to-FFM ratio at 6.6 yr with BMI or FFMI at 7.6 yr.

Greater SLJ-to-BMI ratio at 6.6 yr was associated with lower BMI, FMI, FM% and WHtR at 7.6 yr in unadjusted (all p<0.001) and adjusted models (p = 0.005, p<0.001, p<0.001, p = 0.003, respectively). Greater SLJ-to-BMI ratio at 6.6 yr was associated as well with lower FFMI (p<0.001) at 1-yr follow-up, but only in unadjusted models. Greater SLJ-to-FFM ratio at 6.6 yr was associated with lower BMI, WHtR and FFMI in both investigated models (all p<0.001), and additionally with lower FMI (p<0.001) and FM% (p<0.001) at 7.6 yr after adjusting for confounders.

Greater speed-agility on the 4x10 m shuttle run task at 6.6 yr was associated with lower FMI (p = 0.001) and FM% (p<0.001) at 7.6 yr in adjusted models. No significant associations were noticed between motor fitness at 6.6 yr and other body composition parameters at 1-yr follow-up.

Longer balance time on one-leg balance test at 6.6 yr was associated with lower BMI, FMI, FM%, FFMI and WHtR at 7.6 yr both in unadjusted models (p = 0.002, p = 0.005, p = 0.011, p = 0.006, p = 0.006, respectively), and as well after adjusting for maternal body mass index, maternal educational attainment, child’s sex and age at the measurements (p = 0.003, p<0.001, p<0.001, p<0.001, p = 0.019, respectively).

### Associations of change in physical fitness with change in body composition between the ages of 6.6 yr (kindergarten) and 7.6 yr (school)

Table 3 demonstrates the associations of changes in PF between the time period of 6.6 yr to 7.6 yr with changes in body composition during the latter age ranges. In unadjusted models, increases in the number of laps in 20 m shuttle run test were associated with decreases in FM% (p = 0.003) and FMI (p = 0.009). No associations between the changes in the number of laps in 20 m shuttle run test with changes in other body compositions parameters neither in unadjusted nor adjusted models were detected.

**Table 3:** Associations of change in physical fitness with change in body composition between the ages of 6.6 yr (kindergarten) and 7.6 yr (school).

	Change^*a*^ in physical fitness^*b*^ (x)													
Cardiorespiratory fitness			Grip-to-BMI ratio		Grip-to-FFM ratio		SLJ-to-BMI ratio		SLJ-to-FFM ratio		Motor fitness		Balance	
Change^*a*^ in body composition (y)	β	*P*	β	*P*	β	*P*	β	*P*	β	*P*	β	*P*	β	*P*
BMI (kg/m^2^)														
Unadjusted	-0.109	0.262	-0.398	**<0.001**	-0.244	**0.012**	-0.558	**<0.001**	-0.442	**<0.001**	0.166	0.081	0.012	0.898
Adjusted ^*c*^	-0.010	0.684	-0.594	**0.002**	-0.264	**0.004**	-0.529	**<0.001**	-0.342	**<0.001**	-0.013	**<0.001**	-0.119	**<0.001**
FMI (kg/m^2^)														
Unadjusted	-0.257	**0.009**	-0.220	**0.023**	0.104	0.287	-0.402	**<0.001**	-0.138	0.160	0.125	0.201	0.064	0.513
Adjusted ^*c*^	0.211	0.358	-0.211	0.563	0.097	**0.019**	-0.349	**<0.001**	-0.192	**<0.001**	0.029	**<0.001**	-0.058	**0.002**
FM (%)														
Unadjusted	-0.294	**0.003**	-0.130	0.184	0.217	**0.025**	-0.292	**0.003**	0.006	0.952	0.092	0.349	0.043	0.665
Adjusted ^*c*^	-0.285	0.264	-0.025	0.742	0.179	**0.014**	-0.352	**<0.001**	-0.166	**<0.001**	0.054	**<0.001**	-0.098	**0.002**
FFMI (kg/m^2^)														
Unadjusted	0.069	0.491	-0.358	**<0.001**	-0.442	**<0.001**	-0.417	**<0.001**	-0.498	**<0.001**	0.121	0.217	-0.018	0.858
Adjusted ^*c*^	0.144	0.453	-0.474	**0.048**	-0.410	**<0.001**	-0.439	**<0.001**	-0.399	**<0.001**	-0.061	**<0.001**	-0.128	**0.003**
WHtR														
Unadjusted	-0.161	0.098	-0.141	0.139	0.053	0.590	-0.530	**<0.001**	-0.381	**<0.001**	0.141	0.139	-0.167	0.078
Adjusted ^*c*^	-0.106	0.457	-0.236	0.427	0.080	**0.016**	-0.431	**<0.001**	-0.209	**<0.001**	0.036	**<0.001**	-0.289	**<0.001**

The results are presented as standardized regression coefficient (β), and *P* value are given for each association. *P* < 0.05 was considered statistically significant.

^*a*^The change in physical fitness and body composition between the measurements at 6.6 yr and 7.6 yr. According to Henriksson et al. (31).

^*b*^Physical fitness was measured using 20 m shuttle run test (cardiorespiratory fitness), handgrip strength test (upper body muscular strength normalized to BMI and FFM: grip-to-BMI ratio, grip-to-FFM ratio, respectively), standing long jump test (lower body muscular strength normalized to BMI and FFM: SLJ-to-BMI ratio, SLJ-to-FFM ratio, respectively), 4×10 m shuttle run test (motor fitness), and one-leg stance test (balance) from EUROFIT battery.

^*c*^Adjusted for maternal BMI, maternal educational attainment, child’s sex and age, at the measurements, baseline values of exposures and outcomes.

BMI—body mass index; FFM–fat-free mass; FFMI—fat-free mass index; FM—fat mass; FMI—fat mass index; WHtR–waist-to-height ratio.

Increases in the grip-to-BMI ratio were associated with decreases in BMI and FFMI in unadjusted (all p<0.001) and adjusted models (p = 0.002, p = 0.048, respectively). Only in unadjusted model, increases in the grip-to-BMI ratio were associated with decreases in FMI (p = 0.023). No significant associations were found between the changes in grip-to-BMI ratio with the changes in FM% or WHtR.

As it was the case with the grip-to-BMI ratio, increases in the grip-to-FFM ratio were associated with decreases in BMI and FFMI in unadjusted models (p = 0.012, p<0.001, respectively) and after adjusting for confounders (p = 0.004, p<0.001, respectively). And increases in the grip-to-FFM ratio were associated with increases in FM% in unadjusted (p = 0.025) and adjusted models (p = 0.014), along with increases in FMI (p = 0.019) and WHtR (p = 0.016) after adjusting for confounders.

Increases in SLJ-to-BMI ratio were associated with decreases in BMI, FMI, FM%, WHtR and FFMI in unadjusted (p<0.001, p<0.001, p = 0.003, p<0.001, p<0.001, respectively) and adjusted models (all p<0.001).

Although we did not observe any associations between the changes in SLJ-to-FFM ratio with changes in FMI and FM% in unadjusted models, increases in SLJ-to-FFM ratio were associated with decreases in FMI (p<0.001) and FM% (p<0.001) after adjusting for maternal BMI, maternal educational attainment, child’s sex and age, at the measurements, and baseline values of exposures and outcomes Furthermore, increases in SLJ-to-FFM ratio were associated with decreases in BMI, WHtR and FFMI (all p<0.001) in both investigated models.

Significant associations between the changes in motor fitness with changes in body composition parameters were present only in adjusted models. Improvements in motor fitness were associated with increases in BMI (p<0.001) and FFMI (p<0.001) and decreases in FMI, FM% and WHtR after adjusting for maternal BMI, maternal educational attainment, child’s sex and age, at the measurements, and baseline values of exposures and outcomes (all p<0.001).

Whilst no associations in unadjusted models were identified, improvements in one-leg stance test were associated with decreases in BMI, FMI, FM%, WHtR and FFMI after adjusting for maternal BMI, maternal educational attainment, child’s sex and age, at the measurements, and baseline values of exposures and outcomes (p<0.001, p = 0.002, p = 0.002, p<0.001, p = 0.003, respectively).

### Associations of physical fitness below or above the median at 6.6 yr (kindergarten) with body composition at 7.6 yr (school)

**Fig 1:**
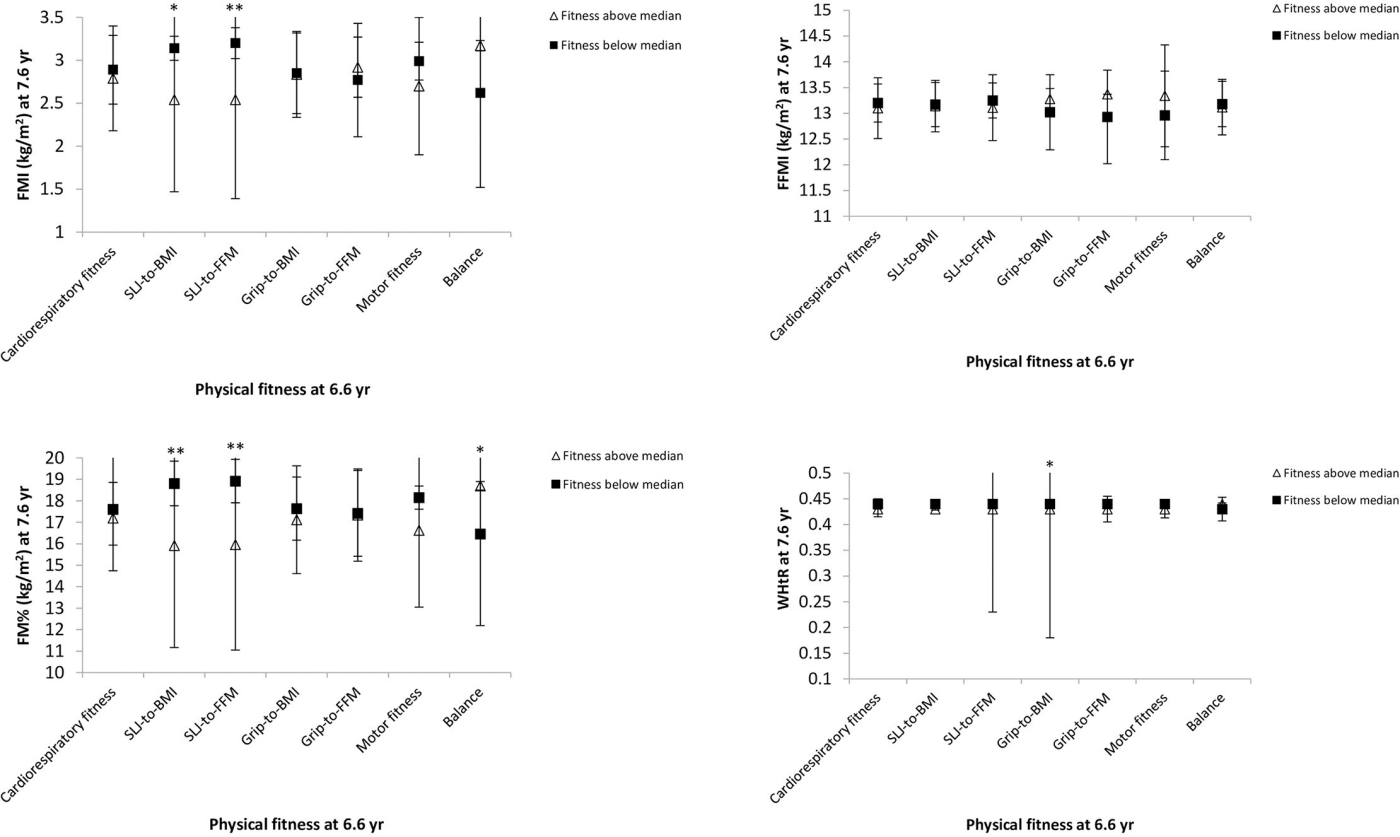
Association of physical fitness below or above the median at 6.6 yr with body composition at 7.6 yr. Analysis of covariance (ANCOVA) was applied adjusting for maternal BMI, maternal educational attainment, child’s age and sex at baseline and follow-up measurements. Data are represented as estimated marginal means with their 95% confidence intervals. BMI—body mass index; FFMI—fat-free mass index; FM—fat mass; FMI—fat mass index; WHtR–waist-to-height ratio. Differences between the two groups: *p < 0.05, **p < 0.01.

Children, whose SLJ-to-BMI ratio and SLJ-to-FFM ratio were above the median at 6.6 yr had lower FMI (p = 0.011, p = 0.003, respectively) and FM% (p = 0.008, p = 0.004, respectively) at 7.6 yr. Additionally, grip-to-BMI ratio above the median at 6.6 yr was associated with lower WHtR (p = 0.036) at 7.6 yr. Balance values above the median at 6.6 yr were associated with greater FMI (p = 0.029) and FM% (p = 0.032) at 7.6 yr. No significant associations between the rest of PF parameters below or above the median at 6.6 yr with body composition at 1-yr follow-up were detected (Fig 1).

## Discussion

This study investigated the associations between physical fitness and body composition in the transition from kindergarten to first grade at school. The main findings of current study were that higher PF at 6.6 yr generally predicted lower BMI, FMI, FM% and WHtR at 7.6 yr among children, who were predominantly in normal weight range (87%). Specifically, better cardiorespiratory fitness, relative upper body muscular strength, motor fitness and balance performance at 6.6 yr were all related to lower FMI and FM% at 1-yr follow-up. Furthermore, greater relative upper and lower body muscular strength along with balance performance at 6.6 yr were associated with lower WHtR at 7.6 yr. We know that metabolic syndrome [5] and type 2 diabetes [6] in children have been mainly linked to excess adiposity, along with potential adverse impact on their psychological well-being [10] and cognitive functions [8]. It has been suggested that more than 50% of PF is determined by environmental factors [33]. It is essential to encourage kids to stay physically active [17, 34] and by improving therefore their PF level, we can help them to develop more healthy body composition with less body fat deposition and healthier WHtR. Fatness and unhealthy body composition have been shown negative associations with the fitness level of children and adolescents in several previous studies [21–23]. The novelty of our study was that 6 yr old children at last year in kindergarten were followed at 1-year later, when they were at first grade in school. In agreement with our results, the cardiorespiratory and motor fitness parameters, and lower body muscular strength at 4.5 yr were negatively associated with FMI and FM% at 5.5 yr in preschoolers [31], greater cardiorespiratory fitness predicted smaller waist circumference among schoolchildren aged 6–15 yr, while low cardiorespiratory fitness was associated with the risk of becoming/remaining overweight [35]. It has been reported that the change in cardiorespiratory fitness of children aged 9.5 yr during 6-yr follow-up period was even stronger predictor of overweight/obesity than childhood fitness, that stresses out the importance of staying physically fit throughout the whole childhood [36]. This was confirmed by Rodrigues et al. [37], who investigated schoolchildren of a similar age at baseline as in the current study during 9 years till the age of 15. The authors concluded that negative developmental pathway in cardiorespiratory fitness (no/small improvements over years) increased several times a chance to become overweight or obese at the end of primary school, compared to children with a positive pathway [37].

Along with cardiorespiratory fitness, other components of PF, such as increased upper and lower body strength and greater speed on 50 m dash test at 6.3 yr were associated with decreased body fat mass during later primary and secondary school years [38]. Our study confirmed the positive effects of both SLJ-to-BMI ratio and SLJ-to-FFM ratio on obesity measures. Specifically, greater relative lower body strength at 6.6 yr predicted lower BMI, FMI, FM% and WHtR at 7.6 yr in adjusted models. Likewise improvements in SLJ test results normalized by BMI or FFM were related to decreases in BMI, FMI, FM% and WHtR during the 12-month follow-up period, after adjusting for maternal BMI, maternal educational attainment, child’s sex and age, at the measurements, and baseline values of exposures and outcomes. In addition, the levels of relative lower body strength above the median at 6.6 yr were associated with lower FMI and FM% at 1-yr follow-up. It seems that better performance on SLJ test was generally associated with smaller body mass, together with muscle mass, if expressed relative to FFM. We detected that SLJ-to-FFM ratio at 6.6 was associated with lower FFMI a year later after controlling for maternal BMI, maternal educational attainment, child’s sex and age, at the measurements, and similarly, its improvement during studied period could be linked to decreases in FFMI after adjusting for maternal BMI, maternal educational attainment, child’s sex and age, at the measurements, and baseline values of exposures and outcomes. Our results are in consistent with suggestions that increased weight status has a negative association with measures of strength involving lifting the body, among children aged 6–15 yr [39].

This study demonstrated that greater handgrip-to-BMI ratio at 6.6 yr was associated with lower FMI and FM% at 7.6 yr which is in agreement with Rodrigues et al. [38]. Likewise, handgrip-to-FFM ratio at 6.6 yr was inversely associated with FM% a year later. However, after adjusting for confounders, the accociations between handgrip-to-BMI ratio with FMI and FM% turned positive. We did not detect any studies to link directly a greater upper body muscular strength with higher FM in children, although increased BMI was negatively associated with measures of strength that involved lifting the body, and conversely, positively associated with performances on tests that did not involve lifting the body, including handgrip strength, among children aged 6–15 yr [39]. It has been speculated about the possibility of increased FM acting as additional training load; as well slow-to-fast skeletal muscle fibre transformation related to lower physical activity in obese, and thus increase in muscle force [40], as well proposed that FM might provide more energy for muscle contractions and thus to support force production [41]. In our study, no associations were found between grip-to-FFM ratio at 6.6 yr with FMI or FM% at 1-yr follow-up. This is in accordance with the work of Henriksson et al. [31], where significant associations between the handgrip strength at 4.5 yr and FMI or FM% at 1-yr follow-up were not detected, after controlling for confounders [31]. Distinct from our study, greater handgrip strength at younger ages was associated with greater FFMI at older age in preschoolers [31]. There are several possible reasons responsible for the discrepancies between studies. Some authors did not apply confounding variables [39], while in our study, the adjustments for potential confounding variables was similar to the previous study of Henriksson et al. [31]. Importantly, to control the maturation, we expressed the handgrip strength as relative to BMI [27, 28] and FFM [30]. In addition, differences in test methodology might have been affected the results. Handgrip strength test is considered the most suitable field test among youth for assessment of upper body muscular strength [42]. We used the handheld dynamometer to assess upper body strength, as did Henriksson et al. [31], but Rodrigues et al. [38] applied the flexed-arm hang test. Henriksson et al. [31] assessed the amount of fat mass and fat-free mass using air displacement plethysmography, while similar to us, Rodrigues et al. [38] performed the analysis based on triceps and subscapular skinfold thicknesses. Children in the study of Henriksson et al. [31] were younger than in our study, that might have had some reflection over the observed differences in outcomes between the studies. If subsequently to analyse longitudinal associations between PF level above or below the median at baseline and body composition parameters a year later, our data show that handgrip-to-BMI values above the median at 6.6 yr were associated with lower WHtR at 7.6 yr. This is consistent with other research, where the waist circumference was assessed in combination with other measures of cardio-metabolic status, and higher levels of muscular strength were associated with lower cardio-metabolic risk in children [43].

No association between the 4x10 m shuttle run test and weight status has been observed in previous studies [38]. Current study agrees with former findings to some point, since before adjusting for confounders, we also did not find any association between the 4x10 m shuttle run results at 6.6 yr and body composition year later, but after controlling for confounders, it appeared that greater speed-agility performance on 4x10 m shuttle run test at 6.6 yr associated with lower FMI, FM% and WHtR at 7.6 yr. Moreover, improved 4x10 m shuttle run test results during the 1-yr follow-up period were related to most healthy body composition parameters as whole (lower FMI, FM%, WHtR and greater FFMI) after controlling for confounders. In fact, the only positive associations related to increases in FFMI in adjusted models were guided by improvements in motor fitness. The positive effect of motor fitness on fat and muscle compartment in adjusted analysis, has been substantiated as well by Henriksson et al. [31].

Balance is considered an important component of motor competence [44], and impaired balance has been associated with increased incidence of injuries [45, 46]. Our results fulfill some gaps in literature about the longitudinal associations between static balance and body composition in children, proposing that better static balance at 6.6 yr predicts lower BMI, FMI, FM%, FFMI and WHtR at 7.6 yr in both regression models. The improvements in one-leg stance test during studied period were also related to decreases in BMI, FMI, FM%, FFMI and WHtR after adjusting for confounders. So far the inverse associations between skinfold thickness or waist circumference and jumping sideward has been demonstrated among younger non-obese children suggesting that children with high fat mass may be less skilled at certain gross motor tasks [47]. Rather surprisingly and contradicting to the results described above, the children, whose balance was better than the median at 6.6 yr had greater FMI and FM% at 7.6 yr in our study. It could be argued that the potential adverse impact of excess body mass on balance will become more apparent among obese children and in case of prevailingly normal weight status the somewhat higher body fat content does not adversely affect the body’s ability to maintain the static balance.

The longitudinal design could be considered as the major strength of the study, because it allows more precise understanding what kind of impact PF status at kindergarten age possesses on body composition later at school. Longer follow-up is needed to clarify the extent of the impact. Another strength of the study is the application of PREFIT and EUROFIT fitness test battery, which are reliable and practically feasible [13, 26, 48]. Finally, the longitudinal associations between static balance and body composition in children are novel, and most of them were advantageous, yet some findings need clearance in further studies.

There are also some limitations of our study that should be mentioned. First, skinfold thickness is described as "midway" marker, that is more sensitive than BMI in calculating body fat [49], and it is assured that Slaughter equation for determining FM% has demonstrated reasonably strong validity with DEXA in children [50]. Since DEXA is considered to be gold standard for measuring body fat composition in children [50], so ideally we suggest this method as the most sensitive one for evaluation of fat and fat-free mass distribution in future studies. Second, we did not measure children’s food intake and energy expenditure, that are important in the context of physical fitness and body composition. In addition, as in the study of Henriksson et al. [31], the participants in current study were predominantly in normal weight ranges, thus the results can not be generalized to overweight/obese population.

## Conclusions

Better PF status in kindegarten will be transmitted towards more favourable changes in body composition at school, expressed by generally lower BMI, FMI, FM% and WHtR among children who are predominantly in normal weight range. Compared to other PF tests, the improvements in 4x10 m shuttle run test results during the 12-month follow-up period were linked to healthy body composition status the most, being the only test that was related to greater FFMI alongside with many other beneficial associations.

Not all studies could find associations between PF with later BMI, relating it to bidirectional effect of FMI and FFMI on BMI and questioning the applicability of BMI in the age group of 4.5 to 5.5 yr [31]. We agree, that suggestions about the relationships of children’s PF with body composition should not be made solely based on BMI, but analysing in complex with the fat and fat-free mass.

The associations between body balance with later body composition parameters appeared generally favourable, still some remain controversial, and to be elucidated in future studies.

Since unhealthy body composition during infancy and early childhood demonstrates risk for obesity and cardiometabolic disease in later life [5], our results emphasise the need to improve children’s PF levels by parents, kindergarten teachers, pediatricians and family physicians already at early age.

## Supporting information

S1 File (SAV)
